# The effect of probiotics on immunogenicity of spermatozoa in couples suffering from recurrent spontaneous abortion

**DOI:** 10.1186/s12865-022-00506-3

**Published:** 2022-06-20

**Authors:** Mitra Rafiee, Nasrin Sereshki, Razieh Alipour, Vahid Ahmadipanah, Davod Pashoutan Sarvar, David Wilkinson

**Affiliations:** 1 Department of Immunology, Cellular and Molecular Research Center, Birjand University of Medical Sciences, Birjand, Iran; 2 Department of Immunology, School of Medicine, Isfahan University of Medical Sciences, Isfahan, Iran; 3 Asadabad School of Medical Sciences, Asadabad, Iran; 4 University of Aberdeen, Scotland, UK

**Keywords:** APCA, HLA class I & II, Probiotic, Spermatozoa, Recurrent spontaneous abortion (RSA)

## Abstract

**Background:**

Impaired spermatozoa immunogenicity can result in pregnancy complications such as recurrent spontaneous abortion (RSA). Given that spermatozoa contact with microbiota, it is possible that inappropriate microbiota composition in the reproductive tract could result in the alteration of spermatozoa antigenicity. Probiotics, as a representative of microbiota, may therefore have a beneficial effect on this altered immunogenicity. The objective of this study was to determine the effect of probiotics on spermatozoa immunogenicity.

**Methods:**

Twenty-five fertile couples and twenty-five RSA couples were included in this study. Spermatozoa were purified and treated with probiotics. Untreated and probiotic treated spermatozoa were evaluated for human leukocyte antigen (HLA) class I & II expression by flow cytometry. Untreated and probiotic treated spermatozoa were also cocultured with the wife’s peripheral blood mononuclear cells (PBMC) for 12 days. Then, the supernatant was assessed for IgG and APCA by enzyme-linked immunosorbent assay (ELISA) and complement-dependent cytotoxicity (CDC) assay respectively.

**Results:**

Probiotic treatment of spermatozoa leads to an increase of HLA class I & II expression in both the fertile and RSA groups. The probiotic treatment resulted in a decrease in both IgG and APCA in the fertile group, but an increase in both IgG and APCA in the RSA group.

**Conclusions:**

The results of this study suggest that a supplementary probiotic treatment may be useful in couples suffering from RSA with an immunologic cause, because it improves disturbed HLA expression on spermatozoa and improves disturbed APCA and IgG production in the presence of spermatozoa.

## Background

There is evidence to suggest that the immune response needed for pregnancy to occur is induced at insemination, and that any impairment in semen and spermatozoa immunogenicity (the ability to stimulate an immune response) can result in pregnancy complications [1]. Recurrent spontaneous abortion (RSA) is related to the alteration of spermatozoa antigenicity [2]. It is well known that semen is in contact with microbiota in the male and female reproductive tracts [3, 4]. There are many findings about the role of microbiota in reproductive health and the immune system, as well as the positive effects of probiotics in improving some reproductive disorders and the immune system [3, 5–7]. Despite these findings, no one, to the best of our knowledge, has studied the effect of microbiota or probiotics on the immunogenicity of spermatozoa.

Probiotics are defined as ‘‘live microorganisms that, when administered in adequate amounts, confer a health benefit on the host” [8]. Recent evidence suggests that probiotics have a beneficial effect on reproduction by altering the microbial ecosystem and the immune response [9, 10]. Lactobacilli that form the majority of the female reproductive tract (FRT) microbiota are the organisms commonly used as probiotics [9]. Several studies have demonstrated the useful effect of probiotics on the prevention of infections in the female reproductive tract and the treatment of common vaginal disorders, namely bacterial vaginosis, candidiasis [9, 11] and preeclampsia [11].

As far as we know, no researchers have addressed the effect of probiotics in RSA. RSA is classically defined as the occurrence of three or more clinically detectable pregnancy losses before 20 weeks of gestation [12]. The etiology of RSA is varied and includes genetic, anatomical, endocrine, immunological, placental anomalies and other factors [12]. Despite numerous studies into the immunologic cause of RSA and the different treatments for this complication, there are still many unresolved questions and controversy about the effectiveness of treatments of RSA with immunologic causes.

Findings reveal that the result of semen and spermatozoa immunogenicity is the development of regulatory and effector T lymphocyte directed to paternal antigens [1, 13, 14] such as human leukocyte antigen (HLA) molecules. Another result of this stimulated immunity may be the production of alloantibody against paternal HLA molecules. Recently, we showed that spermatozoa could induce maternal peripheral blood mononuclear cells (PBMCs) for production of anti-paternal cytotoxic antibody (APCA) [15]. APCA is a pregnancy alloantibody and is directed to paternal HLA molecules that it can be detected in the serum of pregnant women and multiparous women [16]. Several studies have shown the absence or reduction of this antibody in the serum of RSA women [17–20].

Recently, we showed decreased expression of HLA class I & II on spermatozoa in men whose wives suffer from RSA [21]. Therefore, the cause of the absence or reduction of APCA in RSA women may be decreased expression of HLA class I & II by the husband’s spermatozoa. We suppose that the cause of decreased expression of HLA by spermatozoa may be due to the inappropriate composition of microbiota in RSA couples. Because spermatozoa can interact with microbiota via their toll-like receptors (TLRs) [22] and one of the consequences of TLR signaling is the increase of HLA molecules [23], disturbed expression of TLRs can result in the disturbed expression of HLA. According to this assumption, our recent study demonstrated decreased expression of TLR4 by the husband’s spermatozoa in RSA couples. Taken together, we hypothesize that the cause of decreased APCA production in RSA women could be due to the decreased expression of HLA on the husband’s spermatozoa and that probiotics may be able to alter HLA expression and improve APCA production via interaction with spermatozoa.

Therefore, this study aims to evaluate whether probiotics can improve HLA expression and the induction of APCA and immunoglobulin G (IgG) -isotype of APCA-production by spermatozoa. We investigated the effect of Lactobacillus casei rhamnosus Döderleini, a commercially available vaginal probiotic, on spermatozoa immunogenicity, for the first time.

## Results

The results of the Two-Way Mixed ANOVA showed that there was no significant effect of type on each of the variables scores separately, as follows:

- HLA-I: F(1,48) = 806.9, *p* < 0.001, η*p*^2^ = 0.94
- HLA-II: F(1,48) = 438.36, *p* < 0.001, η*p*^2^ = 0.9
- IgG: F(1,48) = 6207, *p* < 0.001, η*p*^2^ = 0.999
- APCA: F(1,48) = 166.65, *p* < 0.001, η*p*^2^ = 0.776

This indicates that the overall mean performing similarly in the fertile group is higher than in the RSA group (Table 1).

**Table 1:** Total mean of the HLA-I, -II, IgG and APCA variables in type group

*HLA-I (%)*			
	Mean	SE	N
Fertile	34.85	0.538	25
RSA	13.22	0.538	25
*HLA-II (%)*			
	Mean	SE	N
Fertile	41.8	0.83	25
RSA	17.22	0.83	25
*IgG concentration (ng/ml)*			
	Mean	SE	N
Fertile	687.46	5.6	25
RSA	56.7	5.6	25
*APCA (%)*			
	Mean	SE	N
Fertile	67.26	1.2	25
RSA	44.48	1.2	25

*N* number, *SE* standard error

In addition, there was also significant main effect of treat for each of the variables separately, as follows:

- HLA-I (F(1,48) = 57.55, *p* < 0.001, η*p*^2^ = 0.546)
- HLA-II (F(1,48) = 63.34, *p* < 0.001, η*p*^2^ = 0.569).
- IgG (F(1,48) = 18.49, *p* < 0.001, η*p*^2^ = 0.28)
- APCA (F(1,48) = 51.59, *p* < 0.001, η*p*^2^ = 0.52)

This indicates that the overall mean for HLA-I and HLA-II in the probiotic treated condition is significantly higher than in the untreated condition, but for IgG and APCA it is significantly lower than in the untreated condition (Table 2).

**Table 2:** Ttotal mean of the HLA-I, -II, IgG and APCA variables in treat groups

*HLA-I (%)*			
	Mean	SE	N
Untreated	21.01	0.532	50
Probiotic treated	27.04	0.566	50
*HLA-II (%)*			
	Mean	SE	N
Untreated	26.52	0.74	50
Probiotic treated	32.5	0.65	50
*IgG concentration (ng/ml)*			
	Mean	SE	N
Untreated	398.88	5.8	50
Probiotic treated	345.2	8.6	50
*APCA (%)*			
	Mean	SE	N
Untreated	60.78	1.01	50
Probiotic treated	50.96	1.2	50

*N* number, *SE* standard error

**Fig. 1:**
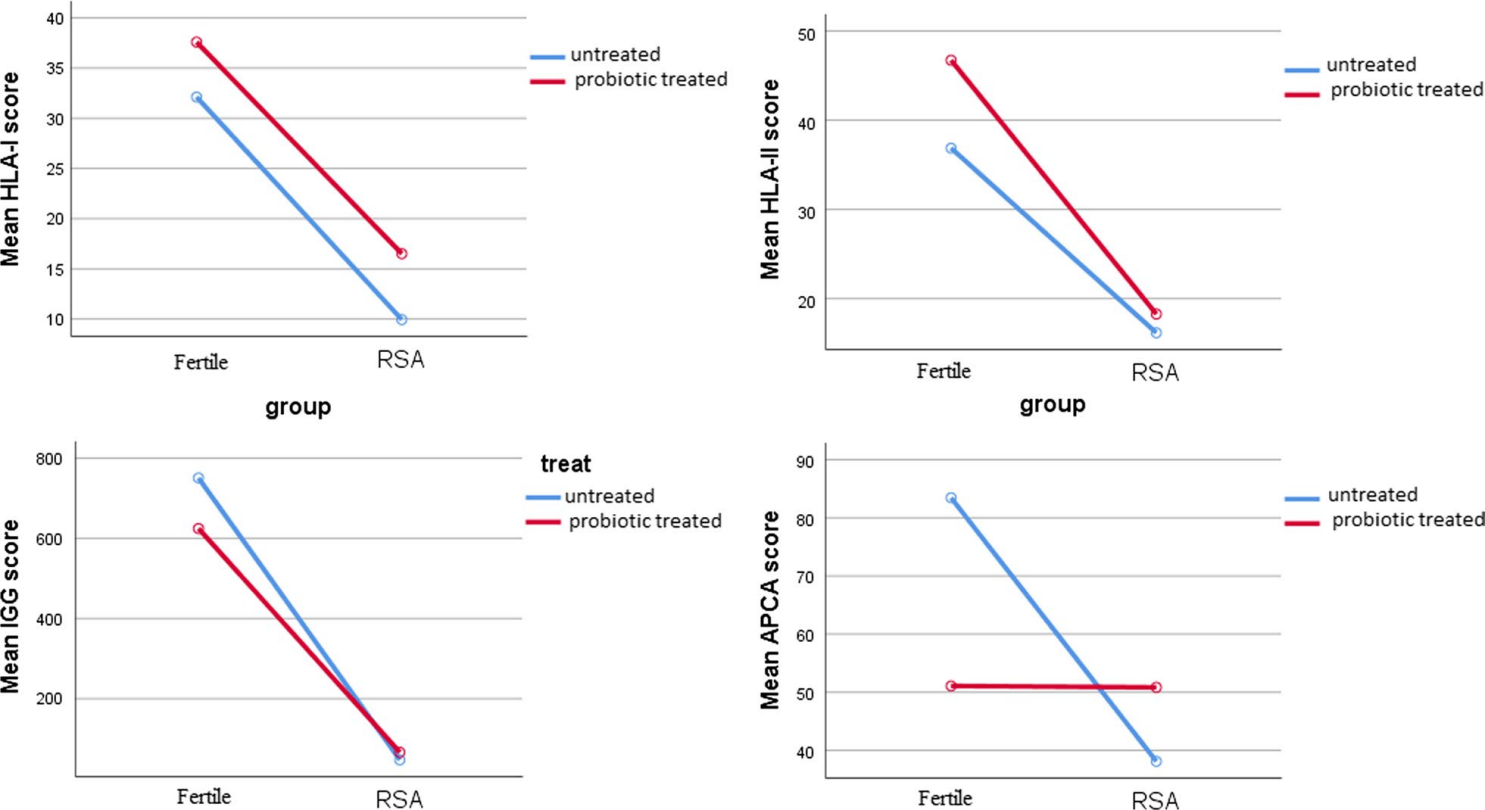
Profile plot of type (fertile, RSA) versus treat (untreated, probiotic treated)

In contrast, there was significant interaction between type and treat for HLA-II (F(1,48) = 26.72, *p* < 0.001, η*p*^2^ = 0.358), IgG (F(1,48) = 33.78, *p* < 0.001, η*p*^2^ = 0.413) and APCA (F(1,48) = 273.71, *p* < 0.001, η*p*^2^ = 0.851), but not significant for HLA-I (F(1,48) = 5.2, *p* = 0.5, η*p*^2^ = 0.01). (Fig. 1).

Bonferroni adjustments were administered for multiple comparisons and the results are summarized in Tables 3 and 4.

**Table 3:** Results obtained from group comparison (untreated and probiotic treated)

		Paired sample t-test			
		Untreated	Probiotic treated	T, P, D	Explanation
HLA-I (%)	Fertile	Mean = 32.11 Sd = 4.8 N = 25	Mean = 37.58 Sd = 5.3 N = 25	T(24) = −3.7 *P* = 0.001 D = 1.04	Significant Probiotic > Untreated
	RSA	Mean = 9.9 Sd = 2.1 N = 25	Mean = 16.51 Sd = 1.9 N = 25	T(24) = −9.8 *P* < 0.001 D = 3.1	Significant Probiotic > Untreated
HLA-II (%)	Fertile	Mean = 36.9 Sd = 6.05 N = 25	Mean = 46.7 Sd = 6.2 N = 25	T(24) = 8.8 *P* < 0.001 D = 1.5	Significant Probiotic > Untreated
	RSA	Mean = 16.17 Sd = 4.2 N = 25	Mean = 18.27 Sd = 1.85 N = 25	T(24) = 2.09 *P* = 0.047 D = 0.62	Non-significant
IgG concentration (ng/ml)	Fertile	Mean = 750.48 Sd = 55.6 N = 25	Mean = 624.44 Sd = 84.25 N = 25	T(24) = 5.1 *P* < 0.001 D = 1.7	Significant Probiotic < Untreated
	RSA	Mean = 47.28 Sd = 19.48 N = 25	Mean = 66.12 Sd = 19.87 N = 25	T(24) = −3.2 *P* = 0.003 D = 0.92	Significant Probiotic > Untreated
APCA (%)	Fertile	Mean = 83.44 Sd = 6.11 N = 25	Mean = 51.08 Sd = 9.46 N = 25	T(24) = 16.3 *P* < 0.001 D = 3.9	Significant Probiotic < Untreated
	RSA	Mean = 38.12 Sd = 8.06 Number = 25	Mean = 50.84 Sd = 7.5 Number = 25	T(24) = −6.8 *P* < 0.001 D = 1.5	Significant Probiotic > Untreated

**Table 4:** Results obtained from group comparison (fertile and RSA)

		Independent sample t-test			
		Fertile	RSA	T, P, D	Explanation
HLA-I (%)	Untreated	Mean = 32.11 Sd = 4.8 N = 25	Mean = 9.9 Sd = 2.1 N = 25	T(32.62) = 20.81 *P* < 0.001 D = 5.9	Significant Fertile > RSA
	Probiotic treated	Mean = 37.58 Sd = 5.3 N = 25	Mean = 16.51 Sd = 1.9 N = 25	T(30.12) = 18.6 *P* < 0.001 D = 5.2	Significant Fertile > RSA
HLA-II (%)	Untreated	Mean = 36.9 Sd = 6.05 N = 25	Mean = 16.17 Sd = 4.2 N = 25	T(48) = 13.97 *P* < 0.001 D = 3.9	Significant Fertile > RSA
	Probiotic treated	Mean = 46.7 Sd = 6.2 N = 25	Mean = 18.27 Sd = 1.85 N = 25	T(28.23) = 21.9 P < 0.001 D = 6.1	Significant Fertile > RSA
IgG concentration (ng/ml)	Untreated	Mean = 750.48 Sd = 55.6 N = 25	Mean = 47.28 Sd = 19.48 N = 25	T(29.8) = 59.62 *P* < 0.001 D = 16.6	Significant Fertile > RSA
	Probiotic treated	Mean = 624.44 Sd = 84.25 N = 25	Mean = 66.12 Sd = 19.87 N = 25	T(26.66) = 32.24 *P* < 0.001 D = 8.9	Significant Fertile > RSA
APCA (%)	Untreated	Mean = 83.44 Sd = 6.11 N = 25	Mean = 38.12 Sd = 8.06 N = 25	T(48) = 22.38 *P* < 0.001 D = 6.2	Significant Fertile > RSA
	Probiotic treated	Mean = 51.08 Sd = 9.46 N = 25	Mean = 50.84 Sd = 7.5 N = 25	T(48) = 0.099 *P* = 0.92 D = 0.02	Non-significant

*Sd* standard deviation, *N* number

## Discussion

Impairment of spermatozoa antigenicity can lead to impaired immunity to paternal antigens and consequently pregnancy complications such as RSA [2, 21]. Impairment of spermatozoa antigenicity may be a result of interaction with inappropriate microbiota composition. Therefore, probiotics may improve this impaired immunity in favor of pregnancy by immunomodulatory effects. In this study, we investigated the effect of Lactobacillus casei rhamnosus Döderleini, a commercially available vaginal probiotic, on spermatozoa immunogenicity in RSA couples compared to fertile couples. This is the first time that this study has been performed.

We showed that the probiotic treatment of spermatozoa results in an increase of HLA class I in the RSA and fertile groups and an increase of HLA class II in the fertile group, but no significant effect on HLA class II in RSA group. In the present study and our recent study [21], we showed that HLA class I & II expression on spermatozoa is decreased in men whose female partners suffered from RSA (RSA couples). The role of HLA on spermatozoa in reproduction has not yet been determined, but we think that the presence of these molecules on spermatozoa is needed to stimulate immunity in the FRT and consequently to achieve successful pregnancy. More study should be performed to investigate this supposition.

The results obtained from coculture of the wife’s PBMC with probiotic treated spermatozoa and antibody production were very interesting. Probiotic treated spermatozoa result in an increase in IgG and APCA production in RSA couples, but a decrease in IgG and APCA production in the fertile group, so after probiotic treatment there is no significant difference between RSA and fertile couples. This finding indicates that probiotics may be a suitable treatment for RSA couples. Moreover, we think this finding can be evidence for immunomodulatory effects of probiotics on spermatozoa immunogenicity. We could not find any study that investigates the effect of probiotic on spermatozoa from RSA couples, whether in vitro or in vivo.

Various clinical trial studies have shown a beneficial effect of probiotics on humoral vaccine responses (production of antibody) [24–28]. Depending on the type of vaccine antigen, strain of probiotic, understudy people and other factors, probiotics helped to increase IgA and IgG in response to the vaccine [29]. This is in contradiction with our result where, in the fertile group, probiotic treated spermatozoa led to a lower level of IgG than untreated spermatozoa. Probiotic treatment of spermatozoa in the RSA group led to increased IgG production accordant to effect of probiotic on vaccine.

Studies have shown that probiotic have a regulatory role on immune response [30–32] and we think this regulatory role of probiotic may be because of the probiotic effect on pattern recognition receptors (PRRs) molecules such as TLR, whatever the further study need. Noteworthy, TLRs play an crucial role in inducing anti-inflammatory or inflammatory response and subsequent activation of adaptive immune responses such as antibody production [33]. We recently showed that decreased TLR4 expression and a differently increased TLR2 expression in response to ligand treatment in spermatozoa is related to unexplained RSA [34]. It is possible that differences in PRR expression or damage-associated molecular pattern (DAMP) expression (self-derived molecules derived from damaged cells that are recognized by PRR) [35–37] on spermatozoa result in abnormal immune stimulation in the FRT and consequently occurring RSA.

Many reports have verified that the amount of TLR trafficking, TLR cleavage, and protein modification of signaling molecules has a major role in activation or inactivation of TLR signaling, and therefore consequent differences in immune response to an antigen [38]. We suppose that decreased antibody production in fertile group and increased antibody production in RSA group, in response to the probiotic, can be a result of differences in TLR expression or TLR response to ligand in RSA group compared to fertile group [34].

It is well known that HLA molecules are the most important antigen in increasing immunogenicity of the cells. Therefore, it is expected that the increase of HLA on spermatozoa results in increased antibody production (IgG/APCA). Expectantly, increase of HLA in RSA group is accompanied with increased antibody production. But unexpectedly in fertile group, increase HLA expression on spermatozoa is accompanied by decreased antibody production. These results raise the question that if probiotic or microbiota have regulatory roles in the expression of different HLA variants with different strength to immunogenicity. Further study should be performed to answer this question. Studies have shown that HLA determinants or allelic variants control antibody production in response to protein antigens [39]. In other words, some alleles of HLA have more capacity to bind and present peptides to T lymphocyte, and consequently have different capacity to induce antibody production [39]. It is possible, there are differences in HLA alleles on the spermatozoa from fertile and RSA groups that, when present peptides of probiotic, result in different capacity in induction of antibody production. Therefore, there is a need to perform a study for determining HLA alleles in spermatozoa from RSA and fertile couples.

Our work clearly has two limitations. The first is the small sample size and the second is the use of a single probiotic strain. However, despite these limitations, this study has gone some way towards investigating immunogenic and immunologic properties of spermatozoa and the interaction of this cell with microbiota.

## Conclusions

The results of this study suggest that a supplementary probiotic treatment may be useful in couples suffering from RSA with an immunologic cause, because it improves disturbed HLA expression on spermatozoa and improves disturbed APCA and IgG production in the presence of spermatozoa and can therefore improve spermatozoa immunogenicity. We hope that our research will serve as a basis for future studies on the immunologic and immunogenic properties of spermatozoa and the interaction of these cells with microbiota.

## Methods

### Ethics statement

The experimental methods were performed in accordance with the relevant guidelines and approved by the Ethics Committee of Asadabad school of Medical Sciences (IR.MUI.REC.1395.3.480). Signed informed consent was obtained from all couples who participated in this study.

### Subjects

Twenty-five fertile couples (women aged 20–40, men aged 22–46) years with at least one child as a control group and twenty-five RSA couples (women aged 25–43, men aged 23–49) with no live births as a case group were included in this study. Noteworthy, during study 10 fertile couples and one RSA couple were excluded from the study because of abnormal semen of husbands. Anti-sperm antibody (ASA) test was negative in these couples. Women under study had no history of blood transfusion or organ transplantation. Women in the control group had no history of any pregnancy complication (e.g., ectopic pregnancy, preterm and post-term labor or preeclampsia). Women in the RSA group, where RSA was due to genetic, anatomical, endocrine, placental anomalies and other factors, were ruled out. The husbands of women in both groups all had normal semen status, according to criteria from the World Health Organization (WHO). Male partners had no history of genital tract disorder such as a history of infection, undescended testis, inguinoscrotal surgery, genital trauma or testicular torsion.

### Purification of Spermatozoa

Semen samples were collected by masturbation after 2–3 days of sexual abstinence. Sampling was performed under sterile conditions. After liquefaction, spermatozoa quality was assessed according to World Health Organization standard guidelines (WHO, 2010). Couples were excluded from the study when the husband had abnormal semen quality. Two ml of AllGrad Wash (LifeGlobal® Group, Canada) were added to the liquefied semen sample and centrifuged at 350 g for 10 min. The pellet was re-suspended in 1 ml of AllGrad Wash. Then in each tube, 1 ml of AllGrad (LifeGlobal® Group, Canada) 90% gradient, followed by 1 ml of AllGrad 45% gradient and then 1 ml of the spermatozoa suspension were carefully layered. After centrifugation at 400 g for 18 min, the pellet was washed with AllGrad Wash and re-suspended in Ham's F-10 medium (dacell, Iran) with 1% bovine serum albumin (BSA) (CMG, Iran).

### Treatment of Spermatozoa

Spermatozoa were seeded at 6 × 10^6^ cells/300 µl in Ham's F-10 medium with 1% BSA in 96-well plates with or without probiotics (Gynophilus vaginal capsules). We used this probiotic capsule because it was abundant in pharmacies and was frequently administered by obstetricians. This commercially available capsule contains Lactobacillus casei rhamnosus Döderleini which are naturally present in the vaginal cavity and lead to the rapid restoration of healthy vaginal flora and the natural pH value in the vagina and also lead to Vaginal flora rich in Lactobacilli and Lactic acid production as a natural protection. One capsule of probiotics was suspended in 1 ml Ham’s F-10 medium and 10 µl of this suspension was added to the wells. It is worth noting that We used this probiotic capsule because it was abundant in pharmacies and frequently, was administered by obstetricians. This commercially available capsule contains Lactobacillus casei rhamnosus Döderleini which are naturally present in the vaginal cavity and leads to the rapid restoration of healthy vaginal flora and the natural pH value in the vagina and also lead to Vaginal flora rich in Lactobacilli and Lactic acid production as a natural protection. Cultures were incubated at 37 °C in a humidified 5% CO2 atmosphere for 4 h. Cultures were performed in duplicate.

### Flow cytometric assay

Untreated and probiotic treated spermatozoa were stained with phycoerythrin (PE) mouse anti-human HLA-ABC (BD pharmingen, USA) and PE-mouse anti-human HLA-DR (BD pharmingen, USA) in distinct tubes. The density of cells was 1 × 10^6^ spermatozoa. After incubation at room temperature for 30 min and two washes with AllGrad Wash (400g for 5 min), spermatozoa were run through the flow cytometer (BD FACS Calibur, USA). Data from at least 100,000 events were collected using forward scatter and side angle of light scatter (a logarithmic amplifier). Fluorescence data were obtained with the logarithmic amplifier. The data were analysed using the FlowJo vx10 software.

### Isolation of peripheral blood mononuclear cells (PBMCs) and performing co-culture

After taking 12 ml heparinized venous blood samples from women under study, peripheral blood mononuclear cells (PBMCs) were separated by centrifugation on a Ficoll-Hypaque (Lymphoprep, Sigma, USA) density gradient. Cells at the interface were harvested, washed twice and were suspended in complete Roswell Park Memorial Institute (RPMI) 1640 medium that was supplemented with HEPES, L-glutamine, penicillin (100U/ml), streptomycin (10 mg/ml), 2-mercaptoethanol (2 × 10^−5^ M) and 20% autologous serum. Two ml of suspension (at the density of 2 × 10^6^ PBMCs) were transferred to the 24-well plates and then cocultured with probiotic treated and untreated spermatozoa at a density of 6 × 10^6^ cells. Cells were then incubated at 37 °C in a humidified 5% CO2 atmosphere. After 4 days, cells were washed 3 times and the pellet was re-suspended in 2 ml complete RPMI 1640 medium, autologous serum was replaced by 20% fetal calf serum (FCS). After 8 days incubation at 37 °C in a humidified 5% CO2 atmosphere, supernatants were harvested, aliquoted and kept in −80 °C until future use.

### Enzyme-linked immunosorbent assay (ELISA)

The concentration of IgG in the supernatant was measured using an enzyme-linked immunosorbent assay (ELISA) kit in accordance with the manufacturer's protocol (IgG (Total) Human Uncoated ELISA Kit, Invitrogen, USA).

### Complement-dependent cytotoxicity (CDC) assay for determining APCA in supernatant

APCA percentage was assessed by cross-matching between supernatants and freshly prepared paternal PBMCs. The test was performed in triplicate in Terasaki plates that were covered with light paraffin oil. One µl of male PBMC suspension (at a density of 2 × 10^3^ cells/ml) was mixed with 1 µl supernatant (1/64 dilution). After 30 min at room temperature, 5 µl rabbit complement (inno-train, Germany) was added. One µl Eosin dye was added to wells after 1 h of incubation at room temperature, followed by 5 µl formalin (37%). The test plates were left overnight to allow the cells to settle. Plates were read using a phase contrast microscope (Olympus, Japan). The number of dead cells among 1000 PBMCs was determined and reported as a percentage of APCA.

Note: in result section, we wrote type and treat group. Type shows fertile and RSA, treat shows untreated and probiotic treated.

### Statistical analyses

Two-factor mixed-design analysis of variance (ANOVA) for type (fertile, RSA), and treat (untreated, probiotic treated) was performed for HLA-I, HLA -II, IgG and APCA. All statistical analyses were conducted using IBM SPSS Statistics for Windows, version 20.0.0.1 (IBM Corp., Armonk, NY, USA). A *P* value of < 0.05 was considered significant. Type show between group and treat wetting group.

## Data Availability

The datasets generated during and/or analyzed during the current study are available from the corresponding author on reasonable request.
